# New York State Veterinary Society

**Published:** 1891-03

**Authors:** N. P. Hinkley

**Affiliations:** Secretary


*New York State Veterinary Society.*—The first Annual Meeting of the New York State Veterinary Medical Society was held at the Parlors of the Vanderbilt House. Syracuse, N. Y. The meeting was called to order by Presd't Morris at 2 P. M. January 16. The following Members responded to the Roll Call by the Secretary Dr. N. P. Hinkley. Drs. Jno. A. Bell, James Carnrite, A. Drinkwater, W. G. Dodds, O. B. French, Wilson Huff, N. P. Hinkley, J. G. Hill, E. D. Hayden, Prof. James Law; Drs. G. H. Moulter, Claude D. Morris, M. M. Poucher, D. K. Seltzer, Robt. Somerville, Harry Sutterby, Frank Sutterby, Jno. Wende. Letters and telegrams of regret were then read from, Prof. Liautard, New York; Prof. Smith, Toronto; Dr. A. L. Aunter, Dr. A. N. McQueen, Dr. Chas Cowie, and several others. President Morris read his Annual Address.

The President asked the Board of Censors to investigate the Credentials of Applicants for Membership. The Chairman of the Board, Dr. H. Sutterby reported in favor of the following gentlemen.

Dr. Louis Robinson, Buffalo; Dr. Wm. Kirk, Niagara Falls; Dr. J. M. Chase, Poplar Ridge; Dr. P. K. Sidebottom, Rochester; Dr. Geo. Gowland, Auburn; Dr. H. S. Wende, Tonawanda; Dr. E. A. Wieland, Buffalo; Dr. Wm. H. Carpenter, Johnstown, N. Y.

On motion of Dr. H. Sutterby seconded by Dr. Drinkwater, the above gentlemen were duly declared elected Members of the Society. The Secretary read the minutes of the last meeting all of which was approved as read. There being no unfinished business the President called upon the Chairman of the Committee of Arrangements for his report. Dr. Hinkley read the report which was duly accepted with thanks. Dr. Sutterby here took occasion to ask the opinion of the Members as to having one day's Session instead of two, at the regular meetings. Giving as a reason that it was taking up too much valuable time. He thought if the members could come to the place of meeting the night before, and call the meeting to order early in the morning, that the busines might by properly done in one day. Dr. Bell opposed and gave good reason for having two days' session. Several members entered into a discussion about making the change. It was put to a vote which resulted unanimously in favor of a one day's session and so declared. Then followed the report of the Committee on Publications which was also accepted.

The report of the Committee on Legislation was called for. The Chairman Dr. C. D. Morris briefly outlined the present so-called Law, and also reported why the proposed Law was defeated at the last session. He also spoke about the lack of knowledge about the Veterinary art of some of the members of the Legislature. Also the want of union among the Veterinary Surgeons. He also read the proposed Act for the benefit of the new members and to have the opinion of all the members expressed. He strongly urged the necessity of prompt and united action of all qualified men in the State of New York and suggested a Committee be appointed by the Society to go to New York and Brooklyn to personally interview the Professional men there and try and get their hearty co-operation in our course. Also for the same committee to appoint two or three members of the committee to remain at Albany to urge the passage of our New Bill. Dr. Carnrite asked the chairman if the facts of how our milk, meat, and dairys were inspected, and the want of Veterinary Surgeons on all Boards of Health, was properly explained to the members of the Legislature last year. Dr. Morris answered that it had been thorougly explained to them. Dr. H. Sutterby asked if it would not be better to ask the co-operation of all Local State Societies to use their influence in getting the proposed Bill passed. President Morris said that was his object in getting a committee to go to New York and Brooklyn, to have a personal interview with the Professional men and to get their views in this matter. Dr. Sidebottom asked for the present Law of registration which was read by the Secretary. Dr. Carnrite asked who was responsible for the present Law. President Morris answered that the present law was framed to put a stop to new unqualified practicing. And in time only qualified men would remain. But the Quacks had brought influence to bear and had the Act extended from year to year.

Dr. Jno. Wende wanted to know what was to prevent the present law from being extended again.

President Morris said that Governor Hill had said it was the last time he would sign the extension. Dr. H. Sutterby moved that proposed Act be printed and a copy placed in the hands of every member of our Society. Also a copy sent to every Veterinarian "Who was a graduate of a Veterinary College or University" in the State of New York. Also to have a fund subscribed by the members of the New York State Veterinary Medical Society to defray the expenses of a Committee to go to New York, Brooklyn and Albany, to assist in getttng the new proposed act passed. The motion was seconded by Dr. Huff and carried.

Dr. H. Sutterby asked for the reading of certain sections of the Bill for the information of some members present who were somewhat in doubt. Dr. Bell asked how many would be needed to go to Albany and about how much money would be required. President Morris said one member would be sufficient. Dr. H. Sutterby said send three members. Dr. J. M. Chase said send two members. D. Darnrite thought there would be no objection to this proposed Act from the Public, but only from unqualified men and their friends Dr. Chase said that in his County there were three graduates and forty-two unqualified men. Dr. Drinkwoter said he had talked with a few prominent unqualified men who said they proposed to ask for a clause in the New Bill to graduate the age of men to come before the Board of Examiners those under the restricted age to be obliged to attend College and graduate before practicing. Dr. W. G. Dodds fully agreed with Dr. Drinkwater regarding the intentions of unqualified men. Dr. Sidebottom cited the English Law on Veterinary practice of qualified men, and Registered men having a right to practice as such.

Dr. Wende said that was just the law we have in New York State at present, the trouble was that the time allowed for registration being extended from year to year. Dr. Hinkley thinks we will have to go slow and get our laws and wants by degrees and to follow in the footsteps of our brother professional men the Medical Doctors, who had been trying a good many years before they had the present law enacted. Dr. Sutterby wanted individual petitions procured and sent to every member in the State. Dr. Gowland thought we must be careful and get the proposed Act into the hands of our friends only. Dr. Carnrite asked if Prof. Liautard assisted in framing the proposed law and if he was in favor of it. Dr. Morris thought that Prof. Liautard was always willing to aid in the passage of any law, or any movement that was made to promote the standing of the qualified Veterinarian and the profession at large, and was certain we could depend upon his assistance.

Several members discussd the feeling between the professional men of the East and West part of the State and all agreed that we should have, *No East, No West*, but one good organization, East and West combined, to protect ourselves. Dr. Carnrite said that even some of the qualified men were acting in company with Quackery, putting up proprietary Medicines and issuing Certificates of Practice to young men who pay them for them. He also stated that if the Public would only investigate they would see that the proposed Bill was more for the protection of the Public than ourselves. Dr. H. Sutterby's original motion was then put to a vote and carried.

Dr. Bell moved that President Morris and Prof. Law be appointed a commitee of two to attend to the passage of the Bill at Albany and to try and get the assistance of Prof. Liautard and other prominent members of the profession to aid. Motion seconded by Dr. John Wende, and unanimously carried. A motion was then made and seconded to adjourn until 8 P. M. to allow members to get supper.

Evening Session, January 16th. Meeting called to order by President Morris, at 8 P. M. The discussion on Legislation was continued. Dr. Bell made a motion that President Morris be made a committee of one to call on members of the profession in New York and Brooklyn; motion was seconded by Prof. Law and carried by unanimous vote.

President Morris then called for the report of the Committee on By-Laws. The report was read by Secretary Hinkley stating that sections 10 and 11, also Code of Ethics and a list of all members had been added to the By-Laws. These were the only changes or additions made up to date. Report accepted and voted on, carried unanimously.

Report on Constitution called for. Owing to absence of the Chairman no report was made. Dr. H. Sutterby made motion to proceed to elect officers for ensuing year. Motion seconded by Dr. Drinkwater, voted on, and carried. The election of officers then took place and resulted as follows: President, Claude D. Morris, V. S., Bath; Vice-President, A. Drinkwater, V. S., Rochester; Secretary, Nelson P. Hinkley, D. V. S., Buffalo; Treasurer, W. D. Dodds, V. S., Canandaigua.

The following were elected a Board of Censors for ensuing year: John Wende, V. S., Buffalo; H. Sutterby, V. S., Batavia; John A. Bell, V. S., Watertown; G. H. Summerfeldt, V. S., Gouvenour; A. L. Hunter, V. S., Watkins.

Dr. Chase made motion, seconded by Dr. Duff, that an adjournment be taken until 9 A. M., January 17th., carried.

Meeting called to order 9 A. M., January 17th, by President Morris, who appointed the following committies to act during ensuing year. Committee on Arrangements: W. H. Carpenter, V. S., Johnstown; P. K. Sidebottom. V. S., Rochester; Geo. Gowland, V. S., Auburn. Committee on Publication: L. A. Robinson, V. S., Buffalo; H. S. Wende, V. S., Tonawanda; Wm. Kirk, V. S., Niagara Falls; E. A. Weiland, V. S., Buffalo; N. P. Hinkley, D. V. S., Buffalo. Committee on Legislation: Prof. James Law, Ithaca; N. P. Hinkley, D. V. S., Buffalo; C. D. Morris, V. S., Bath. Committee on By-Laws: Robert Somerville, V. S., Buffalo; J. M. Chase, V. S., Poplar Ridge; O. B. French, V. S., Honeoye Falls. Committee on Constitution: Frank Sutterby, V. S., Lyons; James Carnrite, V S., Amsterdam; J. G. Hill, V. S., Sennett; M. M. Poucher, V. S., Oswego; Wilson Huff, V. S., Rome.

President Morris then called for the report of the Treasurer and appointed Drs. Chase and Sidebottom, an auditing committee. Report read and accepted by Auditing Committee. The report of the Secretary was then called for, read, and accepted. A motion was made and seconded that an assessment be made on each member, the amount raised to be used toward paying expenses of Legislation. A subscription list was also started and the members present contributed very handsomely, knowing the money raised is to be used for promoting the welfare of the Qualified Veterinarian and procure laws to protect the public at large.

President Morris then called upon Prof. James Law to read his paper.*

A discussion on Prof. Law's paper followed in which all members took an active part. Prof. Law explained how in making his searches and researches with the action of lymph "as prepared by Prof. Koch," and otherwise his subjects being the swine and thoroughbred cattle, both in England and in this country, he had proven that Prof. Koch's experiments were not the first on record.

Dr. A. Drinkwater, of Rochester, then read a paper on "Veterinary Examination as to Soundness of Horses."

A discussion followed in which it was decided by the members present that it was necessary that all qualified Practitioners of the State should adapt a form of Certificate to be given to the owner of horses examined for soundness.

Motion was made and seconded that an adjournment be taken until 2 P. M.; carried unanimously.

Meeting called to order at 2 P. M. Dr. Claude Morris read a paper on "Diseases of the Eye." A very lively discussion followed and several expressed their opinion as to the different modes of treatment in diseases of the eyes, and remedies used.

Dr. John A. Bell then read a paper on Ergotism. Dr. Bell's paper was fully discussed by all members present as was also his mode of treatment in Ergot Poisoning.

---

* Published in the February number of the JOURNAL.

President Morris then called on Dr. John Wende to read his paper. A discussion was entered into on Dr. Wende's paper by all members present and continued until a late hour. A motion was made and seconded that we tender a vote of thanks, and compliment the Essayists for the interest displayed in reading their papers for the benefit of the members and the able manner in which they had prepared them. Carried unanimously.

A motion was made and seconded to adjourn until the semmi-annual meeting to be held in July, 1891. Subject to the call of the Secretary. Carried.

And thus closed one of the most interesting and enthusiastic meetings ever held by the New York State Veterinary Medical Society.

N. P. Hinkley, *Secretary.*

